# Implementing an intra outbreak ring vaccination clinical trial for Sudan Ebola virus disease within four days of outbreak notification in Uganda

**DOI:** 10.1080/22221751.2026.2731496

**Published:** 2026-09-07

**Authors:** Winters Muttamba, Henry Kyobe Bosa, Misaki Wayengera, Andrew Obuku, Maria Sekimpi, Geofrey Kimbugwe, Wilber Sabiiti, Pauline Byakika-Kibwika, Noah Kiwanuka, Rebecca Nantanda, Diana Atwine, Charles Olaro, Diallo Abdourahamane, Alhassane Toure, Ana Maria Henao-Restrepo, Pontiano Kaleebu, Bruce Kirenga

**Affiliations:** a Makerere University Lung Institute, Makerere University, Kampala, Uganda; b Research and Innovation, Interdisciplinary Consortium for Epidemics Research, Kampala, Uganda; c Ministry of Health, Kampala, Uganda; d Makerere University College of Health Sciences, Makerere University, Kampala, Uganda; e Department of Immunology, Uganda Virus Research Institute, Entebbe, Uganda; f Division of Infection and Global Health, School of Medicine, University of St Andrews, Scotland, UK; g Office of the Vice Chancellor, Mbarara University of Science and Technology, Mbarara, Uganda; h Medical Research Council/Uganda Virus Research Institute & London School of Hygiene and Tropical Medicine Uganda Research Unit, Entebbe, Uganda; i Immunology Department, London School of Hygiene and Tropical Medicine, London, UK; j R&D Blueprint, World Health Organisation, Geneva, Switzerland

**Keywords:** Clinical trials, epidemics, Ebola outbreak, vaccine trial, Ebola vaccine trial

## Abstract

East and Central Africa experiences heightened threats for Ebola Virus Disease (EVD), with the latest outbreak caused by the *Bundibugyo ebolavirus* (BDBV) reported in the Democratic Republic of the Congo (DRC) and Uganda on 15th May 2026. The BDBV outbreak comes just over a year following the declaration of Sudan Virus Disease (SVD) outbreak in Uganda on 30th January 2025. Following the 2025 outbreak, a race against the clock mobilization occurred over the subsequent four days to launch a ring vaccination trial for a candidate vaccine against SVD. During this four-day period, the investigation team leveraged ongoing engagements with trial sponsors to activate prefinancing mechanisms and unlock in-kind financing from the government of Uganda. Further, the team activated a prepositioned research protocol to secure rapid ethics and regulatory clearance, and quickly accessed investigation product within the country, supportive ethics/regulatory systems, presence of logistical and data management systems established during the 2022 outbreak, collaborative relationship between the trial and the Ministry of Health outbreak response teams. Finally, previously trained field and community engagement personnel were mobilized overnight, offered refresher training and rapidly deployed to the field. In conclusion, the unprecedented four-day turnaround was made possible by the research, logistical and staffing capacities established during the 2022 SVD outbreak, an achievement that offers a blueprint for rapid evaluation of medical countermeasures during outbreaks.

## Introduction

East and Central Africa experiences heightened threats for Ebola Virus Disease (EVD). The Democratic Republic of the Congo (DRC) and Uganda have had back-to-back outbreaks, with the latest outbreak caused by the *Bundibugyo ebolavirus* (BDBV) announced on 15th May 2026. The BDBV outbreak comes just over a year following declaration of Sudan Virus Disease (SVD) outbreak in Uganda on 30th January 2025.

**Figure 1.**
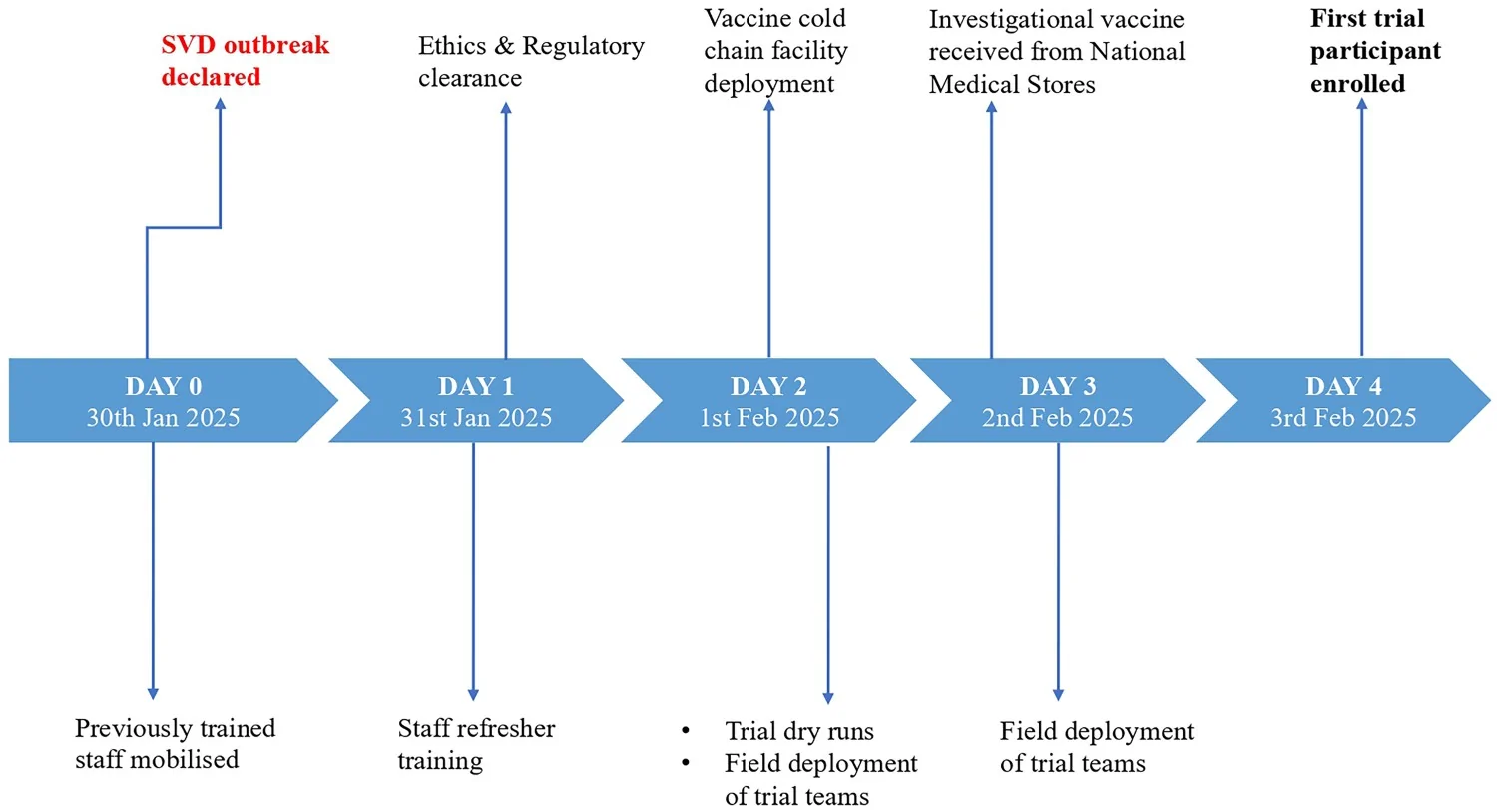
Timeline of ring vaccination clinical trial events.

The back-to-back EVD outbreaks in the region are a testament to the increased frequency of these outbreaks. Equally concerning is that since the first Ebola case was reported in 1976 [1], only one vaccine for Zaire ebolavirus is licenced, while none exists for the Sudan ebolavirus [2]. Following the 2025 outbreak, a race against the clock mobilization occurred over the subsequent four days to launch a ring vaccination trial for a candidate vaccine against SVD (Figure 1). This paper describes the operational breakthroughs achieved within the four-day window, detailing how the investigation team implemented this clinical trial within an unprecedented four days.

## Amplified ongoing engagements with trial sponsors

The investigation team had been in constant contact with trial sponsors (World Health Organization (WHO) and Ugandan Ministry of Health (MOH)) following the 2022 outbreak, where unfortunately the same trial could not be implemented. This was because the outbreak was declared over before participant enrolment could take place. Following the outbreak announcement in 2025, these engagements were immediately amplified. The constant engagement eliminated the need to start communication from scratch.

## Prefinancing mechanism

The implementing institution (Makerere University Lung Institute) proactively sought and obtained approval from the sponsors (WHO and MOH) to prefinance the trial while trial funds were being processed and disbursed by the sponsors. Lack of accessible and timely resources is a common bottleneck that could curtail timely execution of disease outbreak responses, including outbreak-responsive research [3], and this prefinancing mechanism helped overcome the financial delays.

## In-kind financing by the government of Uganda

Within the four days, the team successfully secured the needed in-kind financing from the government through the incident command team at the Ministry of Health. As a result, the trial team was granted access to the isolation centres where some of the potential trial participants were quarantined, allowed use of public health facilities for follow-up visits and use of their waste disposal systems.

## Activating a prepositioned research protocol

A protocol that had been developed and received ethical and regulatory approval in 2022 was used to secure rapid ethics and regulatory clearance. This protocol had been kept as a living protocol and this helped overcome the protracted ethical and regulatory processes that could have delayed trial initiation.

## Timely access to the investigation product and integrity verification

The team successfully secured timely access to the investigational products and ascertained their integrity to ensure immediate field readiness. A vaccine candidate (rVSVΔG-SEBOV-GP) that had been shipped into the country during the 2022 SVD outbreak was kept at the National Medical Stores and the 2434 litre cold chain facility at Makerere University Lung Institute/Mulago Hospital. The integrity of these vaccines was confirmed by collaborating with the manufacturer to audit and verify continuous temperature logs.

## Engagement of ethics/regulatory systems

The team leveraged the long-standing and continuous engagement with the ethics and regulatory boards. Following announcement of the outbreak, these existing channels were intensified to approve the trial for immediate implementation. This engagement also helped the team to amend the ring vaccination protocol and obtain approval to accommodate a sub study to assess the laboratory safety and immunogenicity profile of the candidate vaccine.

## Swift reactivation of logistical and data management systems established during the 2022 outbreak

The team within the four days following the announcement reactivated the systems and structures established during the 2022 SVD outbreak to quickly implement the trial. This capacity has been previously described [4] and included cold chain storage, clinical trial personnel, logistics and data management.

## Rapid operationalization of the collaborative relationship between trial investigators and the Ministry of Health outbreak response teams

The trial framework was immediately embedded directly into the MOH response structures while ensuring the research activities did not interfere with the outbreak response. Ultimately, the trial contributed to outbreak response through identification of contacts, who were also of interest to the response. This close collaboration helped streamline the flow of information, mapping of cases or their contacts and provided the response structures with logistical support.

## Mobilizing pre-trained field and community engagement staff

Field clinical and community engagement professionals trained in good clinical practice and trial protocol during the 2022 SVD outbreak were mobilized overnight, offered refresher training and rapidly deployed. Up to 100 staff were mobilized, divided into 6 field teams and deployed. The immediate activation of the workforce bypassed typical onboarding challenges that would have delayed field deployment.

The implementation of the trial had some challenges. Given the geographical spread of the outbreak, participant samples for laboratory safety and immunogenicity had to be transported over long distances and over difficult geographical terrain to a central processing laboratory at the Uganda Virus Research Institute. This calls for the establishment of mobile research laboratories to ensure timely processing of research samples. Vaccine hesitancy was encountered during field implementation and was addressed through continued community engagement.

## Conclusion

The successful implementation of a ring vaccination trial within an EVD outbreak in an unprecedented four-day period is a testament to operational agility and proactive research capacity building initiatives. The achievement offers a blueprint for rapid evaluation of medical countermeasures in outbreak situations.

## Data Availability

The dataset(s) supporting the conclusions of this article is (are) included within the article.
